# Role of Damage-Associated Molecular Patterns in Septic Acute Kidney Injury, From Injury to Recovery

**DOI:** 10.3389/fimmu.2021.606622

**Published:** 2021-03-01

**Authors:** Pierre-Olivier Ludes, Charles de Roquetaillade, Benjamin Glenn Chousterman, Julien Pottecher, Alexandre Mebazaa

**Affiliations:** ^1^Department of Anesthesiology and Intensive Care, Hautepierre Hospital, Strasbourg University Hospital, Strasbourg, France; ^2^EA 3072, Mitochondrie Stress Oxydant et Protection Musculaire, Faculté de Médecine, FRU 6702, Fédération de Médecine Translationnelle de Strasbourg (FMTS), Strasbourg, France; ^3^Department of Anesthesiology and Critical Care, Hôpital Lariboisière, DMU Parabol, APHP.Nord, Paris, France; ^4^Inserm U942 MASCOT, Université de Paris, Paris, France

**Keywords:** DAMPs, clinical features, precision medicine, kidney recovery, acute kidney injury, sepsis, therapeutic targets

## Abstract

Damage-associated molecular patterns (DAMPs) are a group of immunostimulatory molecules, which take part in inflammatory response after tissue injury. Kidney-specific DAMPs include Tamm-Horsfall glycoprotein, crystals, and uromodulin, released by tubular damage for example. Non-kidney-specific DAMPs include intracellular particles such as nucleus [histones, high-mobility group box 1 protein (HMGB1)] and cytosol parts. DAMPs trigger innate immunity by activating the NRLP3 inflammasome, G-protein coupled class receptors or the Toll-like receptor. Tubular necrosis leads to acute kidney injury (AKI) in either septic, ischemic or toxic conditions. Tubular necrosis releases DAMPs such as histones and HMGB1 and increases vascular permeability, which perpetuates shock and hypoperfusion via Toll Like Receptors. In acute tubular necrosis, intracellular abundance of NADPH may explain a chain reaction where necrosis spreads from cell to cell. The nature AKI in intensive care units does not have preclinical models that meet a variation of blood perfusion or a variation of glomerular filtration within hours before catecholamine infusion. However, the dampening of several DAMPs in AKI could provide organ protection. Research should be focused on the numerous pathophysiological pathways to identify the relative contribution to renal dysfunction. The therapeutic perspectives could be strategies to suppress side effect of DAMPs and to promote renal function regeneration.

## Introduction

### Damage-Associated Molecular Patterns

Damage-associated molecular patterns (DAMPs) are endogenous molecules that are released under various conditions of major cell stress or tissue injury. DAMPs are either exposed on the plasma membrane of stressed cells or actively secreted by stressed cells. They can also be passively released into the cellular environment from dying cells after disruption of their plasma membrane or from the damaged extracellular matrix (1). DAMPs also include homeostatic danger signals, which are associated with perturbations of tissue homeostasis (mechanical stress, hypoxia, and scarcity of nutrients) and may signal a pathological stress (2). A large variety of DAMPs generated from various sources have been described. Several approaches to classify them have been reported, either according to their cell sub-localization or based on their chemical composition (3, 4). A significant body of literature reports on the role of DAMPs in the pathogenesis of acute kidney injury (AKI) and its resolution.

### Acute Kidney Injury

Acute kidney injury is characterized by a rapid decline of renal functions, either glomerular filtration and/or tubular secretion, which leads to an accumulation of metabolic wastes and toxins. AKI can therefore cause distant organ dysfunction, of which, cardiac injury is one the most frequently described (5). The main causes of AKI in clinical settings are sepsis, ischemia-reperfusion injury (IRI), drug-induced nephrotoxicity, and endogenous nephrotoxicity such as rhabdomyolysis. It is estimated that each year two million people die of AKI worldwide and the prevalence has recently increased (6–9). The intensive care unit death rate for AKI is high, ranging from 50 to 80% and a large proportion (30–70%) of surviving patients with AKI can develop complications such as chronic kidney disease (CKD) and end-stage renal disease, possibly requiring long-term dialysis (10).

Inflammation is one of the main triggers for the onset and worsening of AKI. Additionally, many secondary causes of AKI, such as pathogenic bacteria, toxins, ischemia, traumas, and autoimmune or auto-inflammatory disorders, are related to inflammation (11). Tubular cell damage and death are key factors in AKI which lead to tubular damage, inflammation, and vascular dysfunction. Kidney recovery from AKI is characterized by tubular repair and regeneration (12, 13). Loss of renal function due to tubular cell death is associated with the release of DAMPs such as high mobility group box one (HMGB1). These DAMPs stimulate and amplify inflammatory reactions, accelerating tissue damage (14).

In addition to their immediate effects involving innate immunity, DAMPs activate pattern recognition receptors on circulating immune cells. Like their microbial counterparts called pathogen-associated molecular patterns (PAMPs), DAMPs initiate and enhance an immune response by activating toll-like receptors (TLRs) (15, 16). Activation of TLRs triggers antigen-presenting cells (APCs) and enhances antigen presentation by dendritic cells and B cells. Therefore, the mechanism enhances antibody production and promotes kidney diseases such as immune glomerulonephritis immune complex or anti-neutrophil cytoplasmic antibody (ANCA) vasculitis, both of which involve alloimmunity and autoimmunity (15, 17, 18).

In this review, we will first briefly describe the current knowledge of pathophysiology of septic AKI and the most recent knowledge on the crucial role of inflammation in the process of AKI generation and recovery. We will detail the mechanisms of DAMP generation and release during AKI as well as their role in initiating the immune response and triggering loss of kidney function. Finally, we will discuss recent findings on the role of DAMPs in kidney generation after an insult, and we will discuss findings on the therapeutic perspectives that might emerge by attenuating DAMP signaling.

## Pathophysiology of Septic AKI

Pathophysiology of AKI induced by sepsis is an area of intense research. The detailed description has already been reviewed elsewhere (19–21). However, despite decades of intense research, its understanding is still incomplete and constantly evolving. While the hemodynamic nature of AKI has long been the paradigm for sepsis-induced AKI, more recent knowledge has suggested a paradigm shift (22). Although sepsis-induced AKI can demonstrate features of IRI, a key role in acute inflammation and tubular injury has progressively emerged (23–25) with few structural alterations.

### Perfusion Alteration

#### Macrocirculation

Impairment of macrocirculation was initially thought to play a central role in sepsis-induced AKI, as sepsis is most often associated with hemodynamic impairment and shock (26) resulting in impaired kidney perfusion. Still, the evidence to support this hypothesis is very scarce (27), and several animal models of sepsis have revealed that renal blood flow (RBF) was normal or increased during sepsis (22, 28). In these models, AKI developed despite normal or increased RBF, which called for a paradigm shift (29). In humans, the strategy to improve oxygen debt have shown no benefit to mortality rates (27) and use of catecholamine to increase oxygen transport has been associated with increased rates of multiple organ failure and death (30). Similarly, observational studies have shown that prior administration of angiotensin-converting enzyme inhibitors (ACEIs) or administration of angiotensin-receptor blockers (ARBs) among patients with septic shock had no impact on the incidence of AKI or death (31). The same has been observed with the administration of non-steroidal anti-inflammatory drugs (NSAIDs) in a prospective randomized controlled trial (32). This, even though these molecules (ACEIs, ARBs, and NSAIDs) are well-known for their strong hemodynamic effect on blood flow from renal arterioles, i.e., the ACEIs and the ARBs reduce glomerular filtration rate and the NSAIDs reduces RBF.

#### Microvascular and Endothelial Dysfunction

Microvascular dysfunction is defined as a damage to the microvascular cellular components, including endothelial cells, smooth muscle cells, and the pool of circulating blood cells (33).

The deformability of red blood cells is reduced in septic patients, thus coupled with leukocyte activation, these alterations result in an increase aggregability (33). An increased expression of adhesion molecules for cell–cell interaction has been described on the surface of neutrophils of septic patients. Finally, disseminated intravascular coagulation is often encountered in patients with sepsis and contributes to microcirculatory dysfunction (34).

The progressive systemic hemodynamic imbalance during sepsis causes renal microvascular dysfunction and oxygen homeostasis impairment. The oxygen deficit leads to oxidative stress and hypoxemia. There is a reduction of oxygen in renal tissue impairing the production of ATP, needed for Na^+^/K^+^ pump function and Na^+^ reabsorption by the proximal tubule (35). The parenchymal cells switch from aerobic to anaerobic respiration producing reactive oxygen species (ROS). In an aerobic state, ROS are produced by the mitochondria resulting in more cell damage and endothelial cell dysfunction (36). Microvascular dysfunction and the oxidative stress have an important role in sepsis-induced AKI. The association of microvascular dysfunction in the kidney with ROS generation has been studied in the murine cecal ligation and puncture (CLP) model of sepsis (37). Wang et al. have shown an early decrease of mean arterial pressure, RBF, and renal capillary perfusion. These alterations were associated with hypoxia and oxidant generation (37).

These microvascular alterations could occur despite normal RBF (38), suggesting heterogenicity in decreased perfusion of the kidney tissue (39). Swelling of endothelial cells is also a common feature of sepsis-induced AKI. Since the kidney is an encapsulated organ, fluid overload, endothelial swelling, and tissue edema could worsen impaired perfusion in the context of sepsis (40, 41).

### Structural Lesions

#### Cell Death in the Kidney

##### Apoptosis

Apoptosis or programmed cell death normally releases very few DAMPs. Moreover, uptake of apoptotic debris by phagocytic cells generally triggers a rather anti-inflammatory phenotype (42).

Different pathways can initiate this regulated cell death. In the intrinsic pathway, cellular stress leads to the permeabilization of the mitochondrial outer membrane, resulting in the release of apoptogenic factors, including cytochrome c, which then binds Apaf-1 to activate caspase 9 (43). In the extrinsic pathway, ligation of death receptors leads to the recruitment of adapter proteins and the subsequent activation of caspase 8 (44). Stress of the endoplasmic reticulum activates caspase 12 (45) and caspase 2 may be the initiator of apoptosis (46).

However, under several circumstances, apoptotic cells might amplify the inflammatory response and be responsible for further damage (47). Clearance of apoptotic debris is crucial for kidney repair; it is usually managed by monocytes or macrophages in a process called efferocytosis (42). Lack of efferocytosis has been associated with impaired recovery and kidney fibrosis (48). Histological studies have in fact revealed little tubular cell apoptosis in septic AKI (22, 49, 50). Conversely, in an experimental model using human tubular epithelial cells *in vitro*, without hypoperfusion or hypoxia, plasma collected from patients with severe sepsis or septic shock could induce apoptosis and functional alterations in tubular cells and podocytes (51). This observation suggests that mechanisms which trigger apoptosis in the setting of AKI induced by sepsis are soluble in nature.

##### Regulated Necrosis

Added to apoptosis, the regulated necrosis (RN) leads to disruption of membrane integrity. The most critical difference between apoptosis and RN probably lies in the release of DAMPs, which stimulates immunogenicity, a process that is absent in apoptosis (52).

Regulated necrosis defines all the genetically encoded pathways of cell death, which result in the rupture of plasma membrane, regardless of the location of the causative trigger, whether it comes from inside (ferroptosis) or outside the cell (necroptosis and pyroptosis). The terms ferroptosis, necroptosis, and pyroptosis refer to defined RN subroutines. Among these pathways of RN, only necroptosis is involved in sepsis-induced AKI (53).

Ferroptosis consists of necrosis by lipid peroxidation. Labile iron is a known risk factor to develop AKI in clinically relevant settings (54). Today, the definition of ferroptosis can be best characterized by a subroutine of RN, which depends on lipid peroxidation, predominantly mediated by polyunsaturated fatty acids (55).

**Figure 1:**
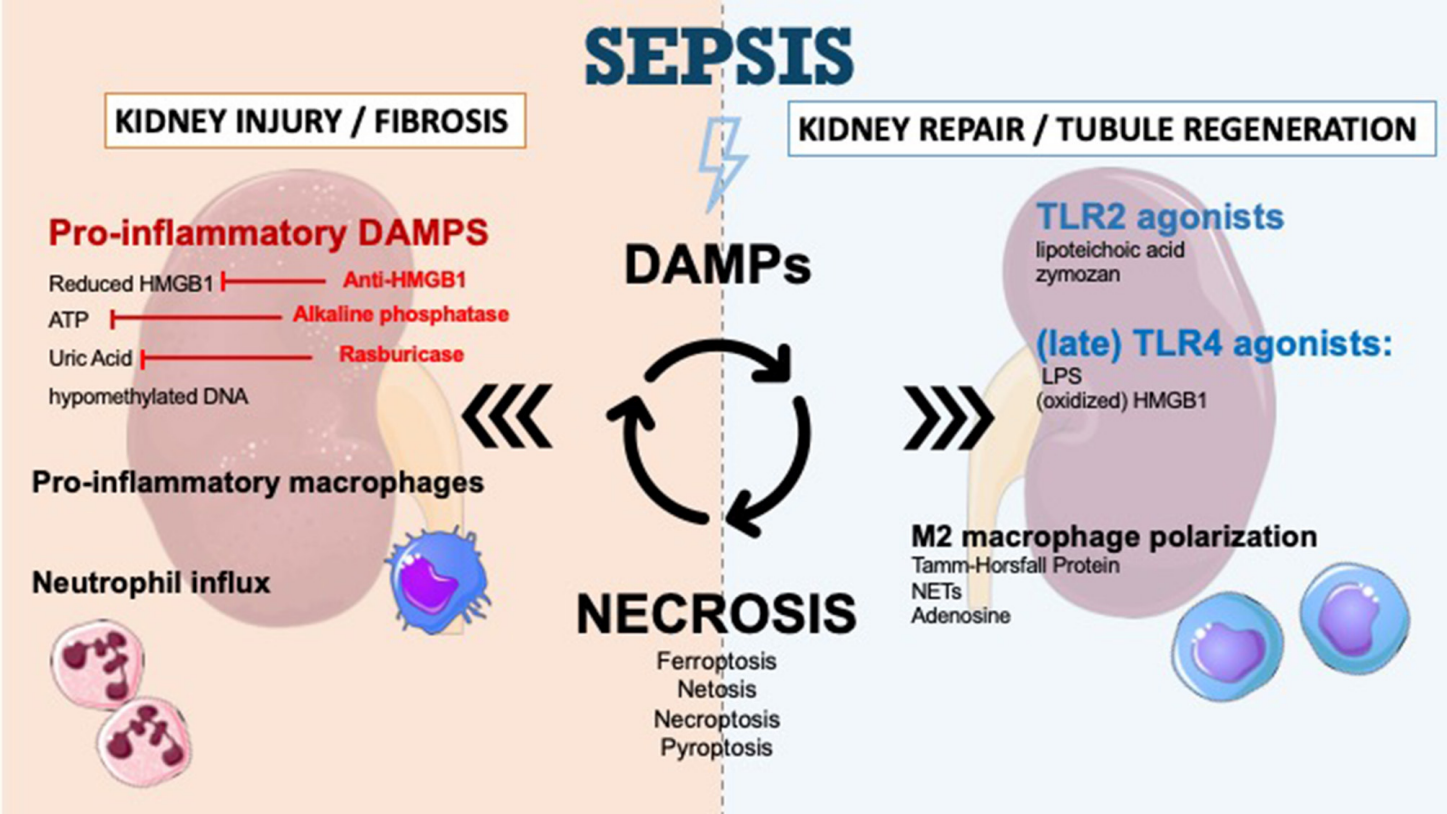
DAMPs in sepsis-induced acute kidney injury and therapeutic perspectives. DAMPs are secreted during sepsis-AKI and enhance kidney injury and kidney fibrosis. The secretion of DAMPs is the result of different mechanisms of necrosis (ferroptosis, necroptosis, pyroptosis, NETosis). Apoptosis leads to very few DAMPs. DAMPs promote immune response and recruit immune cells such as neutrophils to infiltrate the tissue. DAMPs also play a role in tubular regeneration and in restoring kidney function by macrophage polarization and interaction with TLR2/TLR4. The inhibition of specific DAMPs by anti-HMGB1, alkaline phosphatase or Rasburicase could prevent progression of renal dysfunction.

Necroptosis is mediated by a mixed lineage kinase domain-like (MLKL) protein. The activation of MLKL upon its phosphorylation (pMLKL) by the receptor-interacting protein kinase-3 (RIPK3) triggers necroptosis with release of intracellular components (56). MLKL phosphorylation is an essential downstream mediator of necroptosis but not sufficient to cause cell death. Death receptors, such as tumor necrosis factors receptor 1 (TNFR1), and Fas, all belonging to the TNFR superfamily of plasma membrane receptors, may mediate necroptosis (57). Sureshbabu et al. (53) have demonstrated that in mice with polymicrobial septic condition, the RIPK3 promotes sepsis-induced AKI and aggravates kidney tubular injury independently of the classical MLKL-dependent necroptosis pathways (Figure 1). They also observed elevated urinary and plasma RIPK3 levels in patients with sepsis-induced AKI.

Pyroptosis is mediated by gasdermin D, which is cleaved by caspase as well as pro-interleukin-1bêta and pro-interleukin-18. The plasma membrane phosphatidylinositol 4,5-biphosphate (PIP2) is targeted by gasdermin D. Subsequently, membrane extrusion is overwhelming, resulting in extensive bleeding and rupture of the membrane when a critical concentration of PIP2-bound gasdermin D has accumulated at the plasma membrane (58).

#### Tubular Injury

Tubular injury is crucial in sepsis-induced AKI pathophysiology. Tubular cells are prone to microcirculation impairment and inflammation, which makes them particularly vulnerable to sepsis (59). However, in humans, pathological studies have in fact revealed little tubular apoptosis lesions, suggesting that those cells are able to inhibit the processes leading to apoptosis. These cells are able to downregulate their metabolism (60) or undergo cell cycle arrest (61) in order to limit damage and allow repair. Lack of reprogramming after such adaptive response was also linked to subsequent kidney fibrosis and CKD (60, 61).

### Inflammation and Cytokine Storm

Several teams have shown a strong association between the levels of cytokines such as interleukins (IL), especially IL-6 and IL-10, or migration-inhibition factors and the development of sepsis-induced AKI (62). Adhesion and infiltration of leukocytes into kidney tissue appear to be detrimental for kidney function and leads to AKI (63).

Recent experimental data as well as previous histological studies have highlighted the importance of leukocyte recruitment and inflammation as a central actor of sepsis-induced AKI (64). Histological studies revealed that unlike renal IRI, which is characterized by apoptosis or necrosis of tubular epithelial cells, sepsis-induced AKI was characterized by healthy or reversible tubular epithelial cell injury, and occasional necrosis (20, 49). These observations corresponded to a rather conserved architecture with few post-anoxic tubular lesions. These studies have also demonstrated a very strong infiltration of leukocytes, mainly monocytic cells, in the kidney tissue during sepsis (49).

In fact, sepsis is a clinical syndrome characterized by systemic inflammation caused by an infective agent. This process tends to expand and cause multiple organ failure. In particular, AKI results primarily from soluble factors released into the circulation (65). This first event is triggered by cytokines and the massive release of DAMPs and PAMPs into circulation. These DAMPs and PAMPs later trigger and accentuate systemic inflammation (66). Although sublethal injury may be reversible, the death of tubular cells triggers a release of DAMPs which could in turn, amplify inflammation (14). This cascade is called necroinflammation and produces a downward cascade after the primary damage and participates in kidney failure with immune cell infiltration (67). Several studies have pointed out that the level of DAMPs was correlated with survival during sepsis (68, 69).

Damage-associated molecular patterns are central effectors at all levels in the pathophysiology of sepsis-induced AKI. As the propagation medium for inflammation, they contribute to macro/microvascular impairment, endothelial dysfunction, leukocyte recruitment, and tubular injury. This is because they are also capable of perpetuating injury long after the clearance of the bacterial pathogens. In their study on non-human primates, Sursal et al. (70) demonstrated that, conversely to PAMPs (assessed by measurement of circulating bacterial 16S-DNA) which were rapidly cleared, the generation of DAMPs (assessed by the measurement of circulating mitochondrial DNA (mtDNA) continued long after the onset of symptoms. Such observations suggest a central role of DAMPs in the generation and maintenance of systemic inflammatory response syndrome during sepsis (70, 71).

Taken together, those observations highlight the central role of inflammation and leukocyte infiltration in the pathophysiology of AKI during sepsis (62, 72). With their DANGER signal function, DAMPs could play a central role in the process of septic AKI.

## Origin of DAMPs in Septic AKI

Several DAMPs involved in septic AKI are listed in Table 1 with their location and related reference.

**Table 1:** Type of DAMPs in septic AKI.

	**Location**	**Name**	**Interaction**	**References**
Kidney, non-specific	Nucleus	HMGB1	TLR2/TLR4 CXCL12	(73) (74) (76)
		Histones	TLR2/TLR4	(80) (81) (82)
	Mitochondrial	mtDNA	TLR9	(90)
	Extracellular	Decorin	TLR2/TLR4	(87)
		Biglycan	TLR2/TLR4 P2X receptor	(83) (84) (85) (86)
Kidney, specific		Uromodulin	TLR4	(92)

*Several DAMPs (Damage associated molecular patterns) of interest which promote sepsis acute kidney injury and relevant reference. HMGB1, High mobility group box 1; TLR, Toll-like receptor; mtDNA, mitochondrial DNA*.

### Non-kidney Specific DAMPs

#### High Mobility Group Box One

High mobility group box one is an intranuclear protein involved in sepsis and AKI. Once released from stromal or immune cells, it can interact with a variety of cell surface receptors, such as TLR2 and TLR4, leading to various functions. The proinflammatory properties of HMGB1 are by far the most described (73).

High mobility group box one can amplify inflammation by positive feedback during sepsis-induced AKI. HMGB1 is known to be a late-appearing inflammatory cytokine during sepsis. However, when a septic episode complicates a long-lasting chronic kidney disease, HMGB1 is an early appearing cytokine measured in the plasma within 6 h in mice with septic condition due to CLP (74). Observations in the AKI septic mouse model suggest that the renal clearance of HMGB1 is reduced in CKD and that a small release of HMGB1 is enough to induce a significant increase in its plasma concentration. The timing of HMGB1 release therefore depends on the extent of kidney failure predominant at the onset of sepsis (74).

However, it has also been shown that this DAMP could also drive regulatory functions. HMGB1 is a redox sensitive DAMP, containing three cysteines which subject this molecule to redox reactions (75). Depending on the level of oxidation, three isoforms of HMGB1 can coexist at different levels and can trigger different types of immune reactions. Fully reduced HMGB1 ligate with the chemokine CXCL12 which further binds to CXCR4 receptor (76). Conversely, Kazama et al. demonstrated in a mouse model that terminal oxidation of HMGB1, which can be induced by the production of ROS by mitochondria, is sufficient and necessary to inactivate the immunostimulatory effects of HMGB1. The terminal oxidation of HMGB1 may confer to HMGB1 some tolerogenic properties, lowering immune activation following apoptosis (77).

Indeed, while HMGB1 was long believed to be trapped inside the nucleus during apoptosis, it has been shown that oxidized HMGB1 is easily released from the apoptotic cells (77) and triggers tolerance (78). In addition, macrophages can actively release HMGB1 after phagocytosis of apoptotic debris (79), challenging the concept of apoptosis as “the silent death.”

#### Histones

Similar to HMGB1, histones are intracellular and intranuclear proteins, which participate in the compaction of DNA. In addition to their physiological role in homeostatic situations, histones also behave like DAMPs, activating TLR2/TLR4, and inducing local and systemic inflammation (80, 81).

Xu et al. (82) detected histones in the circulation of baboons infected with *Escherichia coli*, and increased histone levels accompanied the development of renal dysfunction. Moreover, these authors have shown that the anti-histone antibody reduced the mortality of mice in lipopolysaccharide (LPS), TNF, and CLP models of sepsis (82).

#### Biglycan

Biglycan is an extracellular DAMP involved in acute ischemic kidney injury and septic AKI, which activates the NRLP3 inflammasome *via* TLR2/TLR4 and P2X receptors (83–85).

Schaefer et al. (85) demonstrated that at the early stage of sepsis induced by LPS and zymosan, the level of TNF-alpha (TNF-α) in serum was considerably lower in biglycan-deficient mice than in wild-type mice. Previously, they observed an overexpression of biglycan and an increase in the number of infiltrating cells in a model of renal inflammation (86).

#### Decorin

Decorin is an extracellular DAMP involved in septic AKI, which activates TLR2/TLR4 (87). Merline et al. measured circulating decorin in plasma samples from a cohort of patients with septic Gram–negative or Gram–positive infections. Immunoblot and ELISA analysis revealed increased levels of decorin protein core in patients with sepsis compared to healthy individuals. The authors experimentally induced sepsis with LPS in decorin-knockout mice, and they showed that the localization of decorin was in close proximity to macrophage in the lungs. In addition, peritoneal macrophages showed enhanced expression of decorin mRNA as well as increased decorin secretion 30 min after LPS stimulation. Nevertheless, the secretion of decorin by renal macrophages should be further demonstrated.

#### Extracellular DNA

Extracellular DNA is a DAMP involved in renal IRI. Necrotic cell-derived DNA leads to platelet activation, platelet-granulocyte interaction, and then neutrophil extracellular trap formation, resulting in renal inflammation and an increase in renal injury (88).

Shi et al. (89) have shown a mouse model of cholesterol crystal (CC) embolism by injection of CC into the kidney artery. The extracellular DNA is involved in CC embolism condition. The arterial obstruction and the organ failure caused by CC embolism is associated with neutrophil extracellular trap formation and DNA release mainly from kidney endothelial cells (89).

More specifically, Tsuji et al. (90) have demonstrated the role of mtDNA in TLR9-associated septic AKI. Their findings on a CLP model of sepsis in wild-type and TLR9-knockout mice suggest that mtDNA activates TLR9 and contributes to cytokine production and kidney injury during polymicrobial sepsis.

### Kidney Specific DAMPs

#### Uromodulin

Uromodulin, also known as Tamm-Horsfall protein, is secreted at the thick ascending limb of the distal tubule. It is an adhesive particle-forming protein, which coats many elements in the distal tubule: cell casts, granular casts (by coating cell debris), and crystal aggregation. The coating of bacteria and inflammatory cytokines also promotes their elimination (91). Inside the tubular lumen, uromodulin is immunologically inactive. However, during tubular injury, uromodulin leaks into the interstitial compartment and becomes a DAMP by activating the interstitial dendritic cells *via* TLR4 (92). In dendritic cells, the NLRP3 inflammasome is activated by phagocytosis and endosomal destabilization in response to the presence of uromodulin in the interstitium. The innate immunity during tubular injury is ultimately activated by the release of IL-1β (93).

## Roles of DAMPs in Kidney Injury and Inflammation

Some studies have shown evidence that microbial proinflammatory agents are among the strongest trigger of inflammation. However, many types of sterile stimuli, including trauma, ischemia, stress, and environmental factors, trigger pathogen-free state of inflammation called “sterile inflammation” (94).

Acute tissue injury triggers a rapid influx of neutrophils. This is followed by increased adhesion of circulating monocyte to activate endothelial surfaces and their subsequent extravasation into interstitial compartments (95). Damaged cells released DAMPs such as HMGB1, ATP, uric acid, or hypomethylated DNA (96, 97). In these tissues, the proinflammatory phenotype of macrophages promotes the secretion of cytokines, chemokines, ROS, and other proinflammatory mediators (98). Proinflammatory macrophages release matrix metalloproteases to enable their migration through basement membranes and interstitial extracellular matrix networks. The resulting small extracellular matrix peptides can function as immunostimulatory DAMPs *via* TLRs and maintain the proinflammatory state (99).

The sterile kidney suffers mainly from infiltrates of proinflammatory macrophages. For example, proinflammatory macrophages release large amounts of TNF-α (100). The link between inflammation and the promotion of kidney injury in sepsis has not been adequately explained. Inhibition of the mechanism of inflammation, such as leucocyte adhesion and infiltration into kidney improves kidney function in sepsis-induced AKI (101). Moreover, in mice with septis-induced AKI, the inhibition of inflammatory cytokines and oxidative stress in dendritic cells and neutrophils improve kidney function (102).

## Roles of DAMPs in Kidney Regeneration and Recovery

Recent data suggest that DAMPs may promote tubule regeneration upon injury and not only to potentiate kidney injury and inflammation (103).

Septic condition leads to inflammation which increases kidney failure and worsens tissue damage; therefore, more necrotic cell parts are released (12, 104). For example, mice lacking HMGB1 are protected from post-ischemic acute renal failure because tubular cell necrosis no longer triggers post-ischemic renal inflammation and tubular damage (105). Nevertheless, recent studies highlighted the role of DAMPs signaling the regeneration post-AKI (106, 107).

### Molecular Mechanisms Leading Recovery From AKI

Recovery from acute tubular injury is not completely understood. This process involves recruitment and repolarization of leukocytes, which are an important source of paracrine growth and signaling factor. The recovery from AKI involves, as a predominant mechanism, an hypertrophy of remnant tubular cells *via* endocycle (108) which is a common cell cycle variant where cells successively duplicate genomic DNA without segregating their chromosome during mitosis (109). The recovery driven by the proliferation and differentiation of progenitor cells appears limited (108, 110). Failure of any of those processes could lead to inadequate repair which could, over time, lead to the development of CKD. Understanding mechanisms leading to AKI-CKD progression is important in order to identify potential therapeutic targets. Therefore, in this process, more attention is paid to the role of DAMPs and their receptors.

#### Role of DAMPs in Monocyte/Macrophages Polarization

During the injury phase, DAMPs and other cytokines prime the M0 monocyte entering a proinflammatory M1 phenotype which contributes to tissue inflammation and perpetuates tissue damage (111). Over time, monocytes evolve toward an anti-inflammatory, M2 phenotype (111). Depletion of macrophages and dendritic cells in the recovery phase is responsible for delayed recovery (112, 113), thus the M1–M2 shift appears to be vital for wound healing and kidney regeneration, promoting tubular cell proliferation and functional recovery (114, 115). The mechanisms leading to the M1–M2 transition are not fully understood but could involve DAMP signaling. Neutrophil extracellular traps (NETs) for example, are thought to induce M2 phenotype (106) while adenosine promotes the shift to anti-inflammatory and angiogenic M2 macrophages by binding to adenosine A2 receptors (Figure 1) (107). In a recent study, Micanovic and colleagues found that the basolateral secreted Tamm-Horsfall protein could trigger M2 macrophage polarization and attenuate inflammation. In their study, THP^−/−^ mice demonstrated aggravated injury and an impaired transition of renal macrophages to M2 phenotype following IRI (116). However, no study to date specifically addressed the role of DAMP signaling in macrophage M2 polarization during septic AKI.

#### Pattern Recognition Receptor

The innate immune system is an important modulator of the inflammatory response during infection and tissue repair. It provides the first line of host defense initiated by several classes of pattern recognition receptors, such as membrane TLRs (117).

##### Agonists of TLR2

Renal progenitor cells (RPCs) have a controversial role in the process of kidney function recovery. Maeshima et al. explored the role of these cells and their involvement in “tubular regeneration concept” after injury. RPCs represent tubular committed progenitors that display resistance to apoptotic stimuli and exert regenerative potential for injured tubular tissue (118). Sallustio et al. (119) have suggested that following insult, TLR2 agonists DAMPs (lipoteichoic acid or zymosan) may induce RPC differentiation and accelerate tubule regeneration (Figure 1). Following the same idea, several studies have reported the use of marrow stromal cells to partially protect the kidney from injury (120, 121). However, the exact role of TLR2 agonists on kidney repair in the context of sepsis remains to be investigated.

The tubular regeneration concept is ruled out by Lazzeri et al. (108). They found that only a few tubular epithelial cells undergo mitosis and contributes to kidney regeneration. Their results indicate that new tubular cells arise exclusively from pre-existing cells that expand, regenerating the entire S3 or distal tubule segment, and therefore behave as progenitor cells.

##### Agonists of TLR4

The role of TLR4 driven signaling in kidney regeneration was recently demonstrated. In an *in vitro* model of IRI, Kulkarni et al. (122) showed that following injury, TLR4-mediated stimulation could induce the release of IL-22 from APCs (dendritic cells and macrophages), which in turn stimulate kidney regeneration. IL-22 is a newly discovered cytokine of the IL-10 superfamily (123) and its involvement in kidney injury and regeneration remains to be studied. Indeed, the exact contribution of IL-22 signaling in the context of septic AKI remains unclear. In a model of peritoneal sepsis, Weber and colleagues demonstrated that blocking IL-22 resulted in reduced organ damage and bacterial load (124). Such a divergence could be explained by different timing of stimulation by IL-22. Indeed, TLR4-mediated stimulation has long be known to play a role in promoting kidney injury during the acute phase (125). Recent reporting has shown that its blockage during the recovery phase can delay tubular recovery (Figure 1) (122). The exact mechanisms by which TLR4 signaling can either mediate inflammation or healing, depending on the timeline, remains to be elucidated.

## Therapeutic Perspectives

Damage-associated molecular patterns can promote and worsen AKI and are involved in the regeneration of renal tissue injury. The modulation of DAMP signaling pathways to control the immune response, as presented in Figure 1, is a field of research.

### Anti-HMGB1

Since extracellular HMGB1 was identified as a late proinflammatory mediator during sepsis, several studies have focused on modulating its signal to decrease sepsis-associated inflammation (126, 127). *In vivo* models of sepsis have demonstrated that HMGB1 could be found 8 h after the onset of sepsis with a peak occurring between 16 and 32 h (128). In a CLP model of sepsis, mice treated with anti-HMGB1 antibody had a significantly lower mortality than mice undergoing CLP alone (129). More importantly, this study demonstrated that late administration of anti-HMGB1 antibodies was able to treat mice with clinical signs of shock, which makes this strategy very interesting in the treatment of human sepsis. Several studies have shown that therapeutic intervention aimed at targeting inflammation induced by HMGB1 could improve survival in animal models (130, 131); however, no study has specifically addressed the potential interest of such an approach in reducing kidney inflammation and the incidence of AKI during sepsis. Still, while anti-HMGB1 therapy has been successful in various clinical conditions, the translation of these promising findings into the clinical field remains to be done.

### Alkaline Phosphatase

Alkaline phosphatase also shows some therapeutic promise in the treatment of sepsis-associated AKI. Alkaline phosphatase is an endogenous enzyme that exerts detoxifying effects by dephosphorylation of both extracellular ATP (132) and endotoxin (133). Recombinant alkaline phosphatase demonstrated its benefit in the preclinical phase (134); however, in a randomized controlled trial enrolling 301 patients, treatment with recombinant alkaline phosphatase did not significantly improve kidney function in the short term (135). More research is needed to clarify the benefits of alkaline phosphatase on other clinical outcomes but also on long-term kidney recovery.

## Rasburicase

Targeting uric acid generation could be of particular interest in the treatment of sepsis-induced AKI.

The mechanisms of uric acid increase in sepsis are unknown and could be due to either increased production or decreased excretion. Severe sepsis may induce ischemia or hypoxia in multiple organs including the kidney and may activate xanthine oxidase in the endothelium to produce uric acid (136).

In fact, under physiological conditions, the majority of uric acid (2/3) is excreted with the urine, while the other third is excreted *via* the intestinal route. Therefore, the release of uric acid could trigger an inflammatory response and worsen kidney injury. In turn, AKI with reduced urine output could increase the flow of uric acid due to lack of urinary excretion (137). Several studies have linked increased plasma uric acid and poor prognosis and reported an increased risk for AKI among patients with elevated uric acid (136, 138). However, in another study using multivariate analysis, such association was no longer significant (139). Uric acid can cause AKI due to a variety of mechanisms ranging from direct tubular toxicity and crystal induced nephropathy to indirect injury secondary to the release of vasoactive mediators and oxidative stress (136). From this perspective, therapy aimed at lowering uric acid during sepsis could be of interest.

## Conclusions

In this review, we aimed to highlight a limited number of recognized DAMPs. We have described the crucial role of these DAMPs in septic AKI as initiating and then amplifying the pathophysiological inflammatory process. Moreover, we stressed the most recent insights in AKI recovery.

In fact, any misplaced or abnormal metabolite level can be a DAMP, and sophisticated analysis studies can reveal the full profile of the DAMPs in numerous conditions included sepsis-induced AKI. Following a major stress, a therapeutic perspective that might emerge is dampening the signaling of DAMPs. This kind of intervention could certainly not be as effective as avoiding the generation of DAMPs. Another objective should be the elimination of the inflammatory agent (140).

## Author Contributions

P-OL, CdR, BGC, JP, and AM conceived of the presented manuscript. All authors discussed the findings and contributed to the final manuscript.

## Conflict of Interest

The authors declare that the research was conducted in the absence of any commercial or financial relationships that could be construed as a potential conflict of interest.
